# Three Novel *KIT* Polymorphisms Found in Horses with White Coat Color Phenotypes

**DOI:** 10.3390/ani15070915

**Published:** 2025-03-22

**Authors:** Nikol A. Obradovic, Aiden McFadden, Katie Martin, Micaela Vierra, Kaitlyn McLoone, Erik Martin, Adelaide Thomas, Robin E. Everts, Samantha A. Brooks, Christa Lafayette

**Affiliations:** 1 Etalon, Inc., Menlo Park, CA 94025, USA; aiden13382@gmail.com (A.M.); khoefs@etalondx.com (K.M.); micaelarvierra@gmail.com (M.V.); kmcloone@etalondx.com (K.M.); emartin@etalondx.com (E.M.); athomas@etalondx.com (A.T.); reverts@etalondx.com (R.E.E.); samantha.brooks@ufl.edu (S.A.B.); 2 Department of Animal Sciences, UF Genetics Institute, University of Florida, Gainesville, FL 32611, USA

**Keywords:** *Dominant White*, depigmentation, *Equus caballus*, mutation, white spotting

## Abstract

This paper reports three novel *KIT* variants likely responsible for previously unexplained white patterning phenotypes observed in three groups of horses. White spots and markings may have substantial consequences on the value and health of domesticated horses. This study aims to elucidate the genetic mechanisms underlying depigmented coat colors to aid in producing prosperous herds. Aligned whole genome sequences were manually screened to identify three polymorphisms in a family of Anglo-Arabian horses (*N* = 7), a family of Warmblood horses (*N* = 5), and a single stock-type mare with unexplained white markings. Sanger sequencing confirmed the presence of the variants, and in silico predictive programs were used to predict the functional impacts of each. We propose to term the novel variants *W37*, *W38*, and *W39*, respectively, per convention. The *W37* polymorphism was always observed in the presence of one *W35* allele, suggesting complete linkage. All three variants were predicted to alter or remove the *KIT* protein active domain, repressing typical protein folding and impacting pathways that upregulate pigmentation. The severe predicted impact on biological function suggests that these variants may cause increased white spotting, providing a possible explanation for the depigmentation phenotypes observed in affected individuals.

## Simple Summary

While more than 50 alleles are associated with white spotting in horses, there remain many instances of white coat patterns with no genetic explanation. Of the previously identified variants, over 35 involve the proto-oncogene *Receptor Tyrosine Kinase* (*KIT*), which is critical for melanocyte survival and proliferation. This study investigated three horse groups with heritable white spotting phenotypes of no known genetic cause. After screening candidate genes previously associated with depigmentation in mammals, we identified three novel variants impacting the coding sequence of *KIT*. Further examination of these variants predicted that the mutations are deleterious to protein function. One novel variant was only observed in the presence of a single *W35* allele, suggesting the alleles may be in complete linkage. We propose to term the novel variants *W37*, *W38*, and *W39* in accordance with standard nomenclature. We report these three novel genetic variants as being likely to cause otherwise unexplained white spotting patterns in horses, and plan future work to examine this association with white spotting in larger populations. Depigmentation is a common phenotype that influences the economic value of a horse; thus, understanding the genetics behind this phenotype is valuable to horse breeders and owners selecting for specific coat color patterns.

## 1. Introduction

While advances in genome sequencing have enabled the detection of genes that influence coat color, the genetic mechanisms behind many white spotting phenotypes in horses remain unknown. White spotting traits frequently impact the commercial and sentimental value of domesticated equids. White markings may determine whether a horse is eligible to enter a breed registry, which strongly affects the economic value of the individual. While some registries select against white markings, others are opening their studbooks to horses with white alleles in an attempt to decrease inbreeding within the population, or may positively select for white spotting phenotypes [1,2,3]. In addition, certain white mutations may lead to deleterious phenotypes, such as sterility or higher risk of deafness or blindness [4,5,6,7,8,9]. For these reasons, detecting the presence of genetic variants that cause white spotting phenotypes has become a principal interest for horse breeders aiming to produce distinctive and healthy herds.

Genetic variations associated with depigmentation (or white spotting) in skin and hair are common in domesticated mammals. In horses, more than 35 of over 50 currently reported white spotting mutations [10] are attributed to mutations in the proto-oncogene, *Receptor Tyrosine Kinase* (*KIT*), as reviewed in McFadden et al. [1]. In mammals, successful melanoblast proliferation and differentiation requires signaling by the *KIT* protein [11,12,13], and some of these variants are predicted to be embryonic lethal in the homozygote [14]. Given that polymorphisms disrupting function or regulation of *KIT* lead to white spotting in humans, mice, and horses, *KIT* is a likely candidate for novel white spotting alleles. Here, we report three novel polymorphisms in *KIT*: a frameshift insertion leading to a premature stop codon, a splice site variant, and a stop-gain variant. This study is a continuation of ongoing research which endeavors to identify the genetic basis for unexplained instances of depigmentation in horses. Building upon previous work in equine genetics, this research expands the existing record of white spotting variants on the *KIT* gene. The findings gathered in this study aim to explain depigmentation in horses belonging to three different breeds.

## 2. Materials and Methods

### 2.1. Horses

Three groups of horses were submitted, with photographs, to Etalon, Inc. for commercial genetic testing. The first family (Family 1) consisted of a half-Arabian, half-Thoroughbred sire and six of his offspring, belonging to the Anglo-Arabian (*n* = 6) and Zangersheide (*n* = 1) breed registries. Two horses, the sire and one offspring, were heterozygous for the *Tobiano* (*TO*) allele and possessed a copy of *Dominant White 35* (*W35*). One offspring was homozygous for *Tobiano*. The other four offspring carried a copy of *W35* but did not have *Tobiano*. All tobiano horses presented large patches of white markings, which is typical of the *TO* variant. The four half-siblings without the *TO* variant displayed white markings on the face and scattered body markings with pink patches of skin and depigmented legs. These four horses exhibited more depigmentation than expected based on their genotypes for other known white spotting alleles.

**Figure 1:**
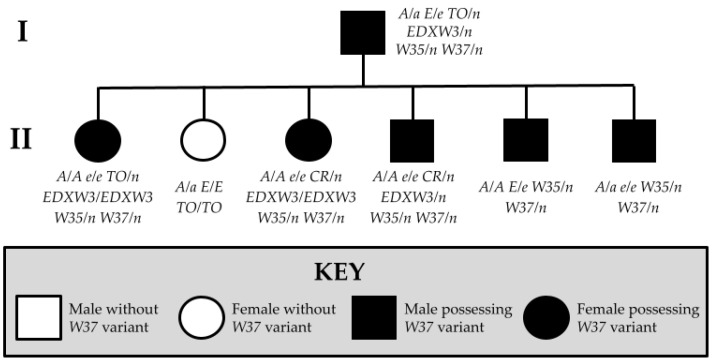
Pedigree of Family 1 displaying the relationships of horses possessing and lacking the novel *W37* variant.

In addition to the *TO* variant, several members of Family 1 possessed copies of the *Eden White 3* (*EDXW3*) allele. Found across diverse horse breeds, *EDXW3* is associated with white spotting phenotypes such as depigmented faces and white legs. Further, horses with two copies of *EDXW3* display an increase in white spotting phenotypes relative to individuals with just one copy of the allele [15]. Two offspring who displayed scattered white markings on their coats possessed neither *TO* nor *EDXW3*. No horses in Family 1 possessed any white spotting alleles other than those explicitly mentioned (Figure 1). 

**Figure 2:**
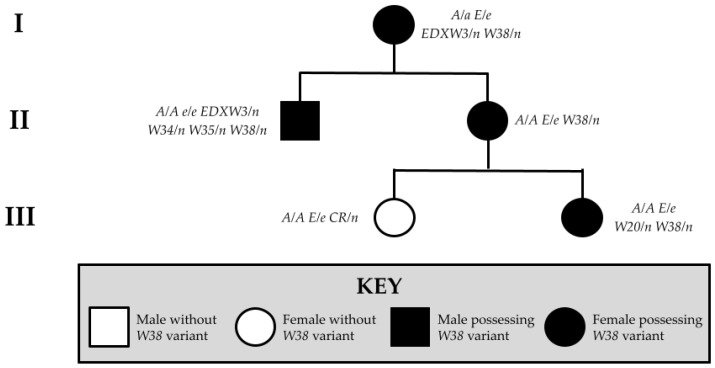
Pedigree of Family 2 displaying the relationships of horses possessing and lacking the novel *W38* variant.

The second family (Family 2) consisted of a Warmblood mare, her dam, her half-sibling, and her two offspring. One of the offspring showed no white markings on her body while the other four family members displayed variable white markings from sabino-like (*n* = 1) to all-white (*n* = 3). The sabino horse carried a single copy of *EDXW3* and no other white alleles. Of the three all-white horses, one carried a single copy of *W20* and another carried a copy of *EDXW3* and *Dominant White 34* (*W34*) in-phase with *W35*. One all-white horse did not carry any known white alleles. None of the horses in Family 2 carried any white spotting alleles other than those explicitly mentioned, nor did they carry the *Grey* (*G*) allele (Figure 2).

A single stock-type mare displaying a nearly all-white phenotype was also investigated in this study. No relatives of this individual were available for phenotyping or genotyping. This mare exhibits some red-pigmented areas of skin and hair limited to her mane, tail, and small patches on the body. Black pigment is retained in all four legs from the fetlock to the knee. No known white spotting alleles or the *Grey* allele were observed in this horse.

### 2.2. Genotyping and Variant Screening

Genomic DNA (gDNA) was extracted from 20 to 30 roots of mane or tail hair using the Puregene Extraction Kit and following the manufacturer’s protocol (QIAGEN, Inc., Germantown, MD, USA). DNA libraries were prepared using the freshly extracted gDNA with xGen Library Preparation Kits, according to the manufacturer’s protocol (Integrated DNA Technologies, Inc., Coralville, IA, USA). Sequences of 150 bp paired-end reads from the NextSeq1000 (Illumina, Inc., San Diego, CA, USA) were aligned to the EquCab3.0 reference genome [16]. The resulting sequences had base call quality scores ≥ Q30 and read depth >40× for all regions under investigation. The binary alignment map (BAM) files produced from the sequencing run were manually screened for novel mutations using NCBI *Equus caballus* Annotation Release 103 gene annotations and the European Variant Archive (EVA) SNP Release 5 on the Integrated Genomics Viewer (IGV) [17,18,19]. Exonic regions of *KIT*, *MITF*, *PAX3*, *ENDRB*, *SOX10*, *LMX1A*, *EDN3*, *HPS5*, *MCOLN3*, *SWAP70*, and *DOCK7* were screened for novel mutations due to the involvement of these genes in pigmentation across diverse mammalian species [1,15,20,21,22,23,24,25,26,27,28,29,30,31,32]. Novel variants were analyzed for predicted significance using the Expert Protein-Analysis System (ExPASy) Translate tool, the Simple Modular Architecture Research Tool (SMART) (version 9), the Iterative Threading ASSEmbly Refinement (I-TASSER) server, and the GENSCAN web server (version 1.0), all with default parameters [33,34,35,36]. Novel variants were confirmed through Sanger sequencing using a commercial service (Azenta Life Sciences, Inc., Burlington, MA, USA).

## 3. Results and Discussion

### 3.1. Novel KIT Insertion Identified in Family 1

**Figure 3:**
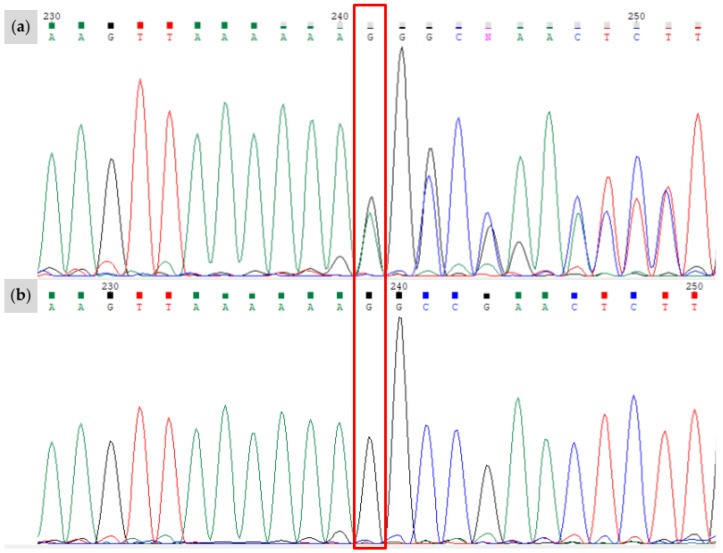
Results of Sanger sequencing for the novel *W37* variant, bounded by the red box. The region shown corresponds to the positive strand of chr3:79,551,893-79,551,914 (EquCab3.0). (**a**) Proband identified as heterozygous for the *W37* allele. The single base pair insertion alters the entire signal downstream of the insertion. (**b**) Offspring of the individual in (**a**) without the *W37* insertion. Sanger sequencing data visualized with Chromas software (version 2.6.6).

After screening candidate genes implicated in white spotting phenotypes, a novel single base pair insertion was identified within the ninth exon of *KIT* (NM_001163866.1) at chr3:79,551,897_79,551,898insA (EquCab3.0). No other novel mutations were identified in the other genes under investigation for Family 1. The novel insertion was not found in a sample of 5334 horses of diverse breeds or in the EVA Release 5 [18]. We found that six of the seven horses from Family 1 possessed the novel insertion, which we term *Dominant White 37* (*W37*). Sanger sequencing results confirmed the presence or absence of the novel insertion (Figure 3).

**Figure 4:**
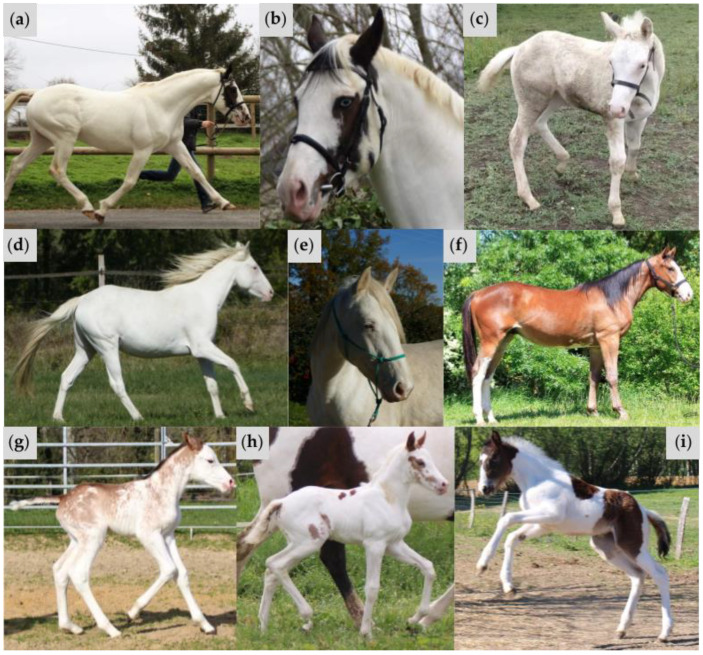
Variable phenotypes of horses in Family 1. Color genotypes are as follows: (**a**) *A/a E/e TO/n EDXW3/n W35/n W37/n*; (**b**) same horse as (**a**), highlighting a blue eye; (**c**) *A/A e/e CR/n EDXW3/EDXW3 W35/n W37/n*; (**d**) *A/A e/e CR/n EDXW3/n W35/n W37/n*; (**e**) same horse as (**d**), highlighting a blue eye; (**f**) *A/A E/e W35/n W37/n*; (**g**) *A/a e/e W35/n W37/n*; (**h**) *A/A e/e TO/n EDXW3/EDXW3 W35/n W37/n*; (**i**) *A/a E/E TO/TO*.

The sire and one offspring had one copy each of *W37*, *W35*, and *TO*, and exhibited a large white patched phenotype, which is often observed in *Tobiano* heterozygotes (Figure 4a,b,g). The remaining four family members were found to have one copy of *W35* and one copy of the *W37* insertion (Figure 4c–f). Horses with the *W37* variant and without *TO* did not demonstrate the entirely white phenotype but displayed sabino-like markings similar to other white spotting mutations involving *KIT* variants. This extensively depigmented abdomen is not a typical phenotype of the *W35* allele. While *W35* is associated with mild white patterning phenotypes ranging from no markings to sabino-like, patches of white hair across the abdomen is a less common trait of the sabino-like horses [1,10]. The sire possessed one blue eye, which is sometimes observed in horses with the *TO* variant. In addition, one offspring with a single copy of the *Cream* (*CR*) allele and the *W37* variant possessed two partial blue eyes. This is not an unusual phenotype for a *Cream* heterozygote, given that the *CR* allele is known to dilute hair, skin, and eye color in horses [6]. The sire and three offspring with the *W37* variant also possessed the *EDXW3* allele, which is associated with increased areas of white spotting, including depigmented faces, lips, and legs. Horses possessing a combination of *W37* with one or more *EDXW3* alleles displayed increased white spotting compared to those with only the *W37* allele.

### 3.2. Novel KIT Splice Site Identified in Family 2

**Figure 5:**
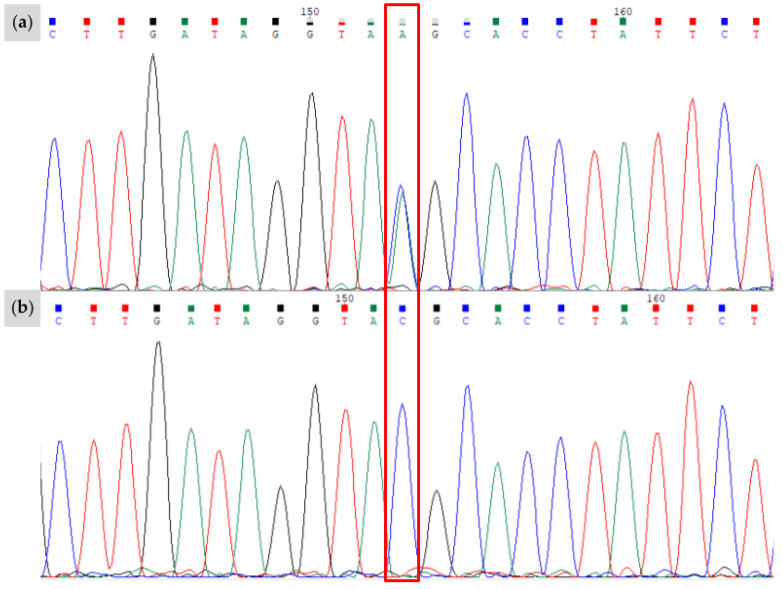
Results of Sanger sequencing for the novel *W38* variant, bounded by the red box. The region shown corresponds to the positive strand of chr3:79,545,856-79,545,878 (EquCab3.0). (**a**) The proband is heterozygous for the “A” allele characterizing *W38*; (**b**) filly of horse in (**a**) without the *W38* variant and genotypes as C/C for this position. Sanger sequencing data visualized with Chromas software (version 2.6.6).

A novel splice site variant in a European Warmblood mare was identified at the +5 base pair of the exon 15 splice donor site of *KIT* (NM_001163866.1, chr3:79,545,867C>A, EquCab3.0). We termed this variant *Dominant White 38* (*W38*) and confirmed its presence with Sanger sequencing (Figure 5). This splice site mutation was not found in a sample of over 5334 horses of various breeds or previously reported in the EVA Release 5 [18]. No other novel mutations were identified in the exonic regions of the other genes examined in this study, nor did any of the horses show the *Grey* allele.

**Figure 6:**
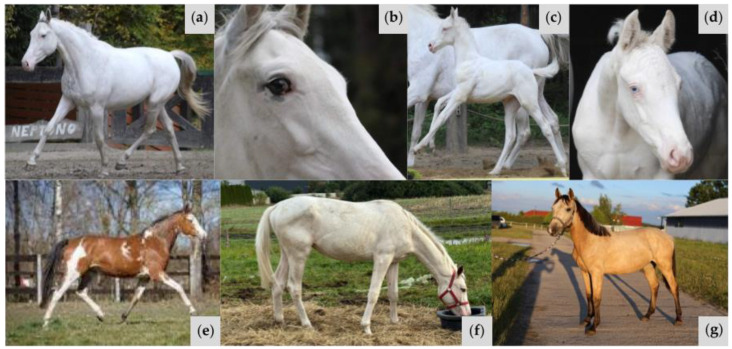
Variable phenotypes of horses in Family 2. Coat color genotypes are as follows: (**a**) *A/A E/e W38/n*; (**b**) same horse as (**a**), highlighting a blue eye; (**c**) *A/A E/e W20/n W38/n*; (**d**) same horse as (**c**), highlighting a blue eye; (**e**) *A/a E/e EDXW3/n W38/n*; (**f**) *A/A e/e EDXW3/n W34/n W35/n W38/n*; (**g**) *A/A E/e CR/n*. Horses possessing the *W38* variant within Family 2 displayed a range of depigmentation, from white markings to an all-white phenotype. The horse in (**e**) exhibits patches of white on the body, high white socks, and a wide blaze, while other family members demonstrate full depigmentation (**a**,**c**,**f**). Depigmentation appears to occur at a greater extent for horses that possess *KIT* variants on the sister copy (compare (**c**,**e**,**f**)).

The proband, one of her offspring, and her half-sibling displayed an entirely white phenotype (Figure 6a,c,f). Both the *W38* proband and one of her offspring possess blue eyes, a less common phenotype of *W* and *EDXW* alleles (Figure 6b,d). The mare’s dam carried one copy of *W38* and one copy of *EDXW3*, displaying a sabino-like phenotype (Figure 6e). One of the horse’s offspring did not demonstrate the all-white phenotype and did not possess the splice site variant (Figure 6g). Two of the all-white horses in Family 2 possess other *KIT* variants out-of-phase with the novel splice site mutation. One of these horses had one copy of *W20*, which was previously reported to increase white spotting in horses with other *KIT* variants [37]. Another all-white horse carried one copy of *W38*, one copy of *EDXW3*, and both *W34* and *W35*, likely also demonstrating a similar phenomenon in which the presence of multiple *KIT* variants enhances the total white spotting phenotype. No variants previously associated with white markings were found in the third all-white horse, so it is currently unclear whether another locus is responsible for the phenotypic differences between this horse and another horse in Family 2 whose phenotype is limited to sabino-like markings (Figure 6a,e). For the *W38* variant, this phenotypic diversity could also be a result of variable *KIT* splicing during development. *KIT* mutations such as *W10* are reported to cause variable degrees of white spotting, even when horses share similar genotypes [1].

### 3.3. Novel Stop-Gain Variant in a Stock-Type Mare

**Figure 7:**
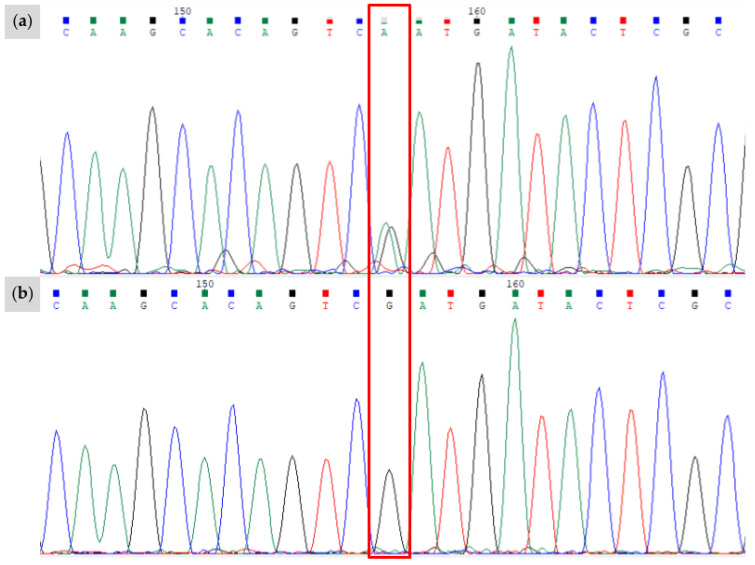
Results of Sanger sequencing for the novel *W39* variant, bounded by the red box. The region corresponds to the positive strand of chr3:79,579,773-79,579,815 (EquCab3.0). (**a**) The proband is heterozygous for the “A” allele characterizing *W38*; (**b**) horse without *W39* and genotypes as G/G. Sanger sequencing data visualized with Chromas software (version 2.6.6).

We identified a novel stop-gain mutation in exon 3 of *KIT*, termed *Dominant White 39* (*W39*), that exchanges an arginine residue at position 181 to a stop codon (NM_001163866.1, chr3:79,579,796G>A, EquCab3.0). The mutation was found in a single stock-type mare, and its presence was confirmed by Sanger sequencing (Figure 7).

**Figure 8:**
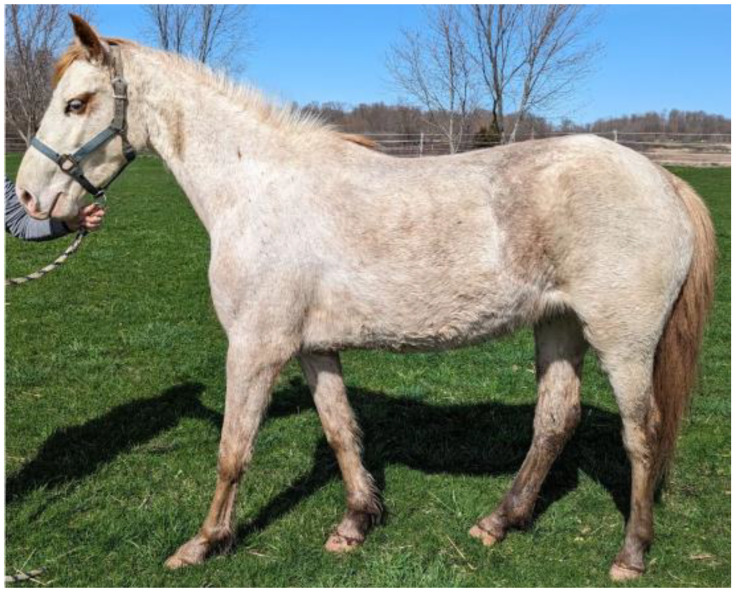
Three-year-old stock-type mare displaying a white spotting phenotype. This horse tested negative for all known white spotting markers yet displays white markings on the face and covering the body with only slight pigment diffused throughout. This individual tested negative for *Cream* and *Grey*.

This three-year-old mare displayed a depigmented body and face. Red pigmentation was retained in the mare’s mane, tail, and small patches on the body. Its four legs were black from the fetlock to the knee, with slight depigmentation in all four pasterns (Figure 8). No overt health defects were reported for this individual. We were unable to source photographs or DNA samples for horses related to this mare, although the owner reported that the dam displayed the same phenotype, so it is unknown how many horses carry this variant. There were no other occurrences of this mutation in a sample group of 5334 other horses of various breeds, nor has it been reported in EVA Release 5. It is likely that the stop-gain mutation is only found within this stock-type horse and her immediate relatives. No other novel mutations or white-causing mutations were found in the exonic regions screened in this study.

### 3.4. Predicted Functional Impacts of Novel Variants

**Figure 9:**
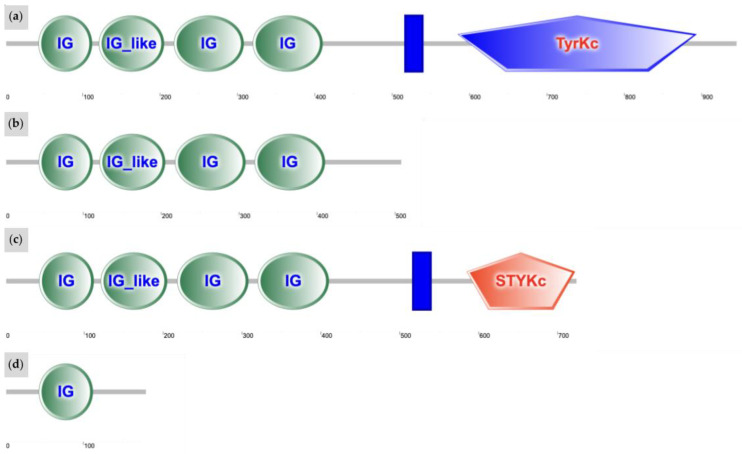
Annotated protein domains for the wild-type *KIT* protein and the predicted changes for novel variants *W37*, *W38*, and *W39*. All mutations (**b**–**d**) impact the active domain, which is predicted to alter catalytic efficiency. (**a**) Domain architecture for the wild-type *KIT* protein, which consists of four IG domains, followed by a transmembrane motif (blue rectangle), and ending with an active tyrosine kinase domain (TyrKc); (**b**) the predicted change in *KIT* domain architecture due to the novel *W37* variant. The protein is prematurely terminated as a result of a stop-gain before the transmembrane domain, resulting in loss of the *KIT* active domain and a loss of specificity for tyrosine; (**c**) in the case of the *W38* variant, a splice site mutation likely causes exon skipping, leading to loss of several kinase elements in the active domain. Consequently, the active domain is altered to a general kinase (STYKc) and loses its affinity for tyrosine; (**d**) for the novel *W39* variant, the protein transcript is truncated after just one IG domain, so no active domain is present within the protein structure. Protein domains generated with SMART software (version 9) [34].

**Figure 10:**
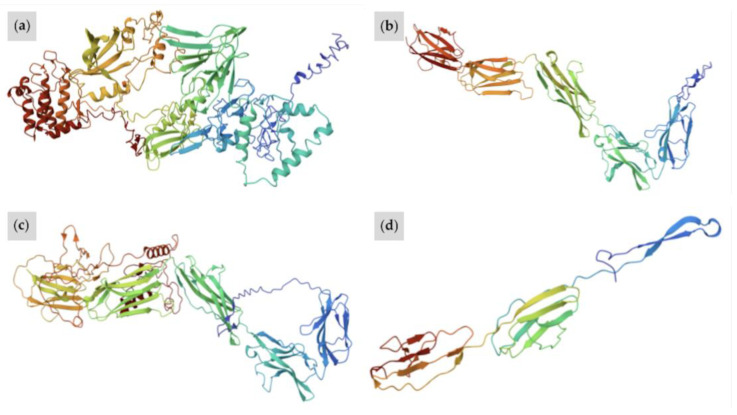
Superimposed protein predictions of (**a**) Wild-type, (**b**) *W37*, (**c**) *W38*, and (**d**) *W39 KIT*. The variants cause alterations of the *KIT* protein active domain that prohibit typical protein folding. All three mutant proteins are more linear in structure than the wild-type, possessing a significantly smaller number of alpha helices and only a number of pleated beta sheets. The predicted structure of the *W37 KIT* protein in (**b**) is the result of an insertion causing a frameshift and leading to a premature stop codon. As this occurs before the active domain, the protein possesses only IG and IG-like domains and is far more linear in structure than its wild-type counterpart. In (**c**), the predicted structure of *W38 KIT* appears the most similar to the wild-type. This mutation is the result of a splice site mutation, which is predicted to slightly alter the active domain and reduce the kinase’s affinity for tyrosine. *W39 KIT* in (**d**) is the most linear protein structure out of all the novel alleles, resulting from truncation of the protein after just a single IG domain. Figures generated with I-TASSER and visualized using Mol* (PDB).

We estimated the functional impact of *W37* on the *KIT* protein using the ExPASy Translate Tool, SMART, and I-TASSER [33,34,35]. The insertion causes a frameshift mutation leading to premature termination of translation. The resulting *W37* protein contains only 504 amino acids compared to the chain of 972 amino acids found in the wild-type protein [17,38]. The SMART-generated prediction of the protein domain architecture demonstrates that the *W37* protein lacks the active kinase domain found in wild-type *KIT* (Figure 9a,b), suggesting that protein function is severely impacted. Since the active kinase domain is downstream of the stop-gain mutation, it would not be translated [39]. The resulting protein would likely lose its selectivity for tyrosine and its enzymatic activity, rendering it incapable of performing signal transduction [11,12,13]. Furthermore, superimposed protein structure predicted by I-TASSER software reveals that the *W37 KIT* protein is significantly more linear in structure compared to wild-type *KIT*. Possessing no alpha helices and fewer pleated beta sheets, the variant represses typical protein folding (Figure 10a,b). The *W37* mutation is therefore likely causative due to its extreme predicted impact on protein function. This is similar to the predicted functional impact observed with the *Dominant White* (*W*) variants *W1*, *W3*, and *W31*. Like *W37*, these variants result from the introduction of a premature stop codon to the translated region of the *KIT* protein and are predicted to critically impact *KIT* receptor function [1].

The functional impact of the *W38* splice site mutation was predicted using the ExPASy Translate Tool, GENSCAN, SMART, and I-TASSER. Mutations at the +5 donor splice site may result in one or more skipped exons [40,41]. The predicted change in *KIT* structure due to *W38* could also permit similar instances of exon skipping [36]. The mutant mature mRNA transcript would be 248 amino acids short of the normal 972 residues. The altered protein would feature just 724 residues with only the first 709 aligning to wild-type *KIT* [33,36,42]. The SMART server predicted that the splice variant would convert the active tyrosine kinase domain to a general kinase [34] (Figure 9a,c). This would decrease specificity for tyrosine and likely disrupt pathways that upregulate pigmentation [11,12,13]. Furthermore, the predicted catalytic domain shows an alteration in the primary, secondary, and tertiary structure relative to the wild-type, suggesting that protein function is impacted [35] (Figure 10a,c). Similarly to *W7*, *W8*, *W11*, and *W13*, this variant would likely inhibit translation of critical domains, leading to impaired melanocyte development and an increase in white spotting [1].

Of all three novel variants, the most severe predicted impact on protein structure is observed in the case of *W39*. Protein architecture predicted by SMART reveals that the stop-gain variant truncates the encoding protein after a single immunoglobulin (IG) domain, removing the entire active kinase domain (Figure 9a,d). In addition, I-TASSER software demonstrates that *W39 KIT* is the most linear of all the superimposed protein structures (Figure 10d). The lack of structural complexity observed in the I-TASSER server prediction suggests the ability of the altered protein to perform its biological function is significantly impaired [35]. This mutation is most similar to *W12*, a small deletion of five bases in exon 3, resulting in the truncation of the encoding protein. The *W12* proband exhibited approximately 50% depigmentation of the body, four white stockings, and a white blaze covering the muzzle [43]. By contrast, the *W39* proband displays nearly full depigmentation of body and face. Some red and black pigment is retained in the mane, tail, legs, and small patches on the body.

A summary of the genotypes, phenotypes, and predicted effects for novel variants for all individuals examined in this study is provided in Table 1. While the populations of horses bearing these phenotypes is small, small sample size is a common attribute of studies that investigate spontaneous and rare alleles in diverse species, including horses and humans. Spontaneous equine W alleles are frequently observed in only the founding individual and its descendants [1]. Furthermore, in a database of 5334 other horses spanning diverse breeds, there have been no additional occurrences of these three novel variants. To analyze the impact of the polymorphisms on *KIT* protein function, this study deferred to guidelines for the interpretation of sequence variants set by the American College of Medical Genetics and Genomics (ACMG). In accordance with ACMG recommendations, multiple in silico predictive programs were utilized as evidence supporting disrupted gene function [44].

Future work could expand the database population to test for the presence of these polymorphisms in a broader and more global sample. Due to the small number of births within these families, further investigation is required to conclude embryonic lethality for each of the three variants. Additional investigation of the combined effects and interactions between the novel alleles and previously identified depigmentation variants is warranted.

## 4. Conclusions

Three novel equine *KIT* variants were identified and based on the predicted functional impacts, each is a strong candidate to explain the observed white spotting phenotypes. A novel insertion, designated *W37*, was identified in Family 1. Horses carrying this frameshift mutation exhibited varying degrees of white spotting. Every horse with *W37* had one copy of *W35*, suggesting the alleles are likely in-phase. A novel splice site mutation, termed *W38*, was discovered in Family 2 horses showing a completely white or sabino-like phenotype. The only offspring without this mutation did not display a sabino-like or all-white phenotype. A stop-gain mutation in exon 3 of *KIT* termed *W39* was discovered in a single mare who demonstrated a nearly all-white phenotype. The W37, *W38*, and *W39* alleles all result in truncated transcripts predicted to alter or remove the kinase domain of *KIT*. Therefore, all novel variants are likely to cause the white spotting patterns observed in the three horse groups under investigation.

Although homozygosity was not observed for any mutation, too few births have occurred to conclude embryonic lethality for any of the three novel alleles. Nevertheless, nonviable homozygosity may be likely due to observed consequences of other similar *KIT* polymorphisms. These variants were not observed in other breeds and were not found in other horses in our sample set of 5334 horses. Future undertakings would expand the number of tested individuals across diverse horse breeds to a global dataset enabling a more comprehensive analysis of the association of the three variants to white patterning phenotypes.

## Figures and Tables

**Table 1:** Individual genotypes, associated phenotypes, and predicted functional impact for novel variants *W37*, *W38*, and *W39*.

Novel Variant	Relationship of Individual to Proband	Genotype	Associated Phenotype	Predicted Functional Impact
*W37*	Proband (Sire) (Figure 4a,b)	*A/a E/e TO/n EDXW3/n W35/n W37/n*	Near all-white with small patches of black pigment on face; one blue eye	Truncation of *KIT* protein prior to active kinase domain inhibits signal transduction processes Typical protein folding is repressed; *W37 KIT* is more linear in structure than wild-type *KIT*, featuring no alpha helices and fewer pleated beta sheets
	Offspring 1 (Figure 4c)	*A/A e/e CR/n EDXW3/EDXW3 W35/n W37/n*	All-white; pink skin	
	Offspring 2 (Figure 4d,e)	*A/A e/e CR/n EDXW3/n W35/n W37/n*	All-white; pink skin; two partial blue eyes	
	Offspring 3 (Figure 4f)	*A/A E/e W35/n W37/n*	Bay with partial bald face and sabino-like depigmentation observed on belly and hind legs	
	Offspring 4 (Figure 4g)	*A/a e/e W35/n W37/n*	Full bald face with extensive sabino-like depigmentation observed on body and legs with red pigment on upper body, mane, and tail	
	Offspring 5 (Figure 4h)	*A/A e/e TO/n EDXW3/EDXW3 W35/n W37/n*	Near all-white with small patches of red pigmentation on body and face	
	Offspring 6 (Figure 4i)	*A/a E/E TO/TO*	Bay with large white patches on body, typical of *Tobiano*	
*W38*	Proband (Figure 6a,b)	*A/A E/e W38/n*	All-white; pink skin with small pigmented spots on skin of body and face; at least one blue eye	Truncated *KIT* protein with conversion of active tyrosine kinase domain to a general kinase; decreased specificity for tyrosine disrupts pathways upregulating pigmentation *W38 KIT* lacks structural complexity of wild-type protein, with fewer alpha helices and only a number of pleated beta sheets
	Offspring 1 (Figure 6c,d)	*A/A E/e W20/n W38/n*	All-white; pink skin; at least one blue eye	
	Dam (Figure 6e)	*A/a E/e EDXW3/n W38/n*	Sabino-like with patches of white on body, high white socks, and wide blaze	
	Half-sibling (Figure 6f)	*A/A e/e EDXW3/n W34/n W35/n W38/n*	All-white; pink skin	
	Offspring 2 (Figure 6g)	*A/A E/e CR/n*	Diluted coat; minimal white marking on muzzle	
*W39*	Proband (Figure 8)	*A/a e/e W39/n*	Roan-like with red hair mixed in with the white hair on body, mane, and tail; Full bald face with small spots of red pigment; black pigment below knees and hocks	*KIT* protein is truncated after just one IG domain and active domain is lost, impairing biological function *W39 KIT* is the most linear of all superimposed proteins, with no alpha helices and few pleated beta sheets

## Data Availability

Horse sequencing data are not available due to owner confidentiality.

## References

[1] McFadden A., Vierra M., Martin K., Brooks S.A., Everts R.E., Lafayette C. (2024). Spotting the pattern: A review on white coat color in the domestic horse. Animals.

[2] Brooks S.A., Palermo K.M., Khan A., Hein J. (2020). Impact of white-spotting alleles, including W20, on phenotype in the American Paint Horse. Anim. Genet..

[3] Santschi E.M., Vrotsos P.D., Purdy A.K., Mikelson J.R. (2001). Incidence of the endothelin receptor B mutation that causes lethal white foal syndrome in white-patterned horses. Am. J. Vet. Res..

[4] Hauswirth R., Haase B., Blatter M., Brooks S.A., Burger D., Drögemüller C., Gerber V., Henke D., Janda J., Jude R. (2012). Mutations in MITF and PAX3 cause “splashed white” and other white spotting phenotypes in horses. PLoS Genet..

[5] Henkel J., Lafayette C., Brooks S.A., Martin K., Patterson Rosa L., Cook D., Jagannathan V., Leeb T. (2019). Whole-genome sequencing reveals a large deletion in the MITF gene in horses with white spotted coat color and increased risk of deafness. Anim. Genet..

[6] Bailey E., Brooks S.A. (2020). Horse Genetics.

[7] Bellone R.R., Holl H., Setaluri V., Devi S., Maddodi N., Archer S., Sandmeyer L., Ludwig A., Foerster D., Pruvost M. (2013). Evidence for a retroviral insertion in TRPM1 as the cause of congenital stationary night blindness and leopard complex spotting in the horse. PLoS ONE.

[8] Rockwell H., Mack M., Famula T., Sandmeyer L., Bauer B.S., Dwyer A., Lassline M., Beeson S., Archer S., McCue M. (2020). Genetic investigation of equine recurrent uveitis in Appaloosa horses. Anim. Genet..

[9] Sandmeyer L.S., Kingsley N.B., Walder C., Archer S., Leis M.L., Bellone R.R., Bauer B.S. (2020). Risk factors for equine recurrent uveitis in a population of Appaloosa horses in western Canada. Vet. Ophthalmol..

[10] Online Mendelian Inheritance in Animals (OMIA). https://omia.org/.

[11] Blume-Jensen P., Claesson-Welsh L., Siegbahn A., Zsebo K.M., Westermark B., Heldin C.H. (1991). Activation of the human c-kit product by ligand-induced dimerization mediates circular actin reorganization and chemotaxis. EMBO J..

[12] Phung B., Sun J., Schepsky A., Steingrimsson E., Rönnstrand L. (2011). C-KIT signaling depends on microphthalmia-associated transcription factor for effects on cell proliferation. PLoS ONE.

[13] Steel K.P., Davidson D.R., Jackson I.J. (1992). TRP-2/DT, a new early melanoblast marker, shows that steel growth factor (c-kit ligand) is a survival factor. Development.

[14] Pulos W.L., Hutt F.B. (1969). Lethal dominant white in horses. J. Hered..

[15] McFadden A., Martin K., Vierra M., Robilliard H., Lundquist E., Everts R.E., Brooks S.A., Lafayette C. (2023). Three Hps5 mutations associated with depigmentation in diverse horse breeds. Livest. Sci..

[16] Kalbfleisch T.S., Rice E.S., DePriest M.S., Walenz B.P., Hestand M.S., Vermeesch J.R., O′Connell B.L., Fiddes I.T., Vershinina A.O., Saremi N.F. (2018). Improved reference genome for the domestic horse increases assembly contiguity and composition. Commun. Biol..

[17] Pruitt K.D., Brown G.R., Hiatt S.M., Thibaud-Nissen F., Astashyn A., Ermolaeva O., Farrell C.M., Hart J., Landrum M.J., McGarvey K.M. (2014). RefSeq: An update on mammalian reference sequences. Nucleic Acids Res..

[18] Cezard T., Cunningham F., Hunt S.E., Koylass B., Kumar N., Saunders G., Shen A., Silva A.F., Tsukanov K., Venkataraman S. (2022). The European Variation Archive: A FAIR resource of genomic variation for all species. Nucleic Acids Res..

[19] Robinson J.T., Thorvaldsdóttir H., Winckler W., Guttman M., Lander E.S., Getz G., Mesirov J.P. (2011). Integrative genomics viewer. Nat. Biotechnol..

[20] Bennett D.C., Lamoreux M.L. (2003). The color loci of mice—A genetic century. Pigment Cell Res..

[21] Magdesian K.G., Tanaka J., Bellone R.R. (2020). A de novo MITF deletion explains a novel splashed white phenotype in an American Paint Horse. J. Hered..

[22] Patterson Rosa L., Martin K., Vierra M., Foster G., Brooks S.A., Lafayette C. (2022). Non-frameshift deletion on MITF is associated with a novel splashed white spotting pattern in horses (*Equus caballus*). Anim. Genet..

[23] Ebanks J.P., Wickett R.R., Boissy R.E. (2009). Mechanisms regulating skin pigmentation: The rise and fall of complexion coloration. Int. J. Mol. Sci..

[24] Hauswirth R., Jude R., Haase B., Bellone R.R., Archer S., Holl H., Brooks S.A., Tozaki T., Penedo M.C.T., Rieder S. (2014). Novel variants in the KIT and PAX3 genes in horses with white-spotted coat colour phenotypes. Anim. Genet..

[25] Metallinos D.L., Bowling A.T., Rine J. (1998). A missense mutation in the endothelin-B receptor gene is associated with Lethal white foal syndrome: An equine version of Hirschsprung disease. Mamm. Genome.

[26] Wang H.H., Chen H.S., Li H.B., Zhang H., Mei L.Y., He C.F., Wang X.W., Men M.C., Jiang L., Liao X.B. (2014). Identification and functional analysis of a novel mutation in the SOX10 gene associated with Waardenburg syndrome type IV. Gene.

[27] Chandra Mohan S.L.N. (2018). Case of Waardenburg Shah syndrome in a family with review of literature. J. Otol..

[28] Steffes G., Lorente-Cánovas B., Pearson S., Brooker R.H., Spiden S., Kiernan A.E., Guénet J.L., Steel K.P. (2012). Mutanlallemand (mtl) and belly spot and deadness (bsd) are two new mutations of Lmx1a causing severe cochlear and vestibular defects. PLoS ONE.

[29] Baynash A.G., Hosoda K., Giaid A., Richardson J.A., Emoto N., Hammer R.E., Yanagisawa M. (1994). Interaction of endothelin-3 with endothelin-B receptor is essential for development of epidermal melanocytes and enteric neurons. Cell.

[30] Di Palma F., Belyantseva I.A., Kim H.J., Vogt T.F., Kachar B., Noben-Trauth K. (2002). Mutations in Mcoln3 associated with deafness and pigmentation defects in varitint-waddler (Va) mice. Proc. Natl. Acad. Sci. USA.

[31] Shinohara M., Terada Y., Iwamatsu A., Shinohara A., Mochizuki N., Higuchi M., Gotoh Y., Ihara S., Nagata S., Itoh H. (2002). SWAP-70 is a guanine-nucleotide-exchange factor that mediates signalling of membrane ruffling. Nature.

[32] Blasius A.L., Brandl K., Crozat K., Xia Y., Khovananth K., Krebs P., Smart N.G., Zampolli A., Ruggeri Z.M., Beutler B.A. (2009). Mice with mutations of Dock7 have generalized hypopigmentation and white-spotting but show normal neurological function. Proc. Natl. Acad. Sci. USA.

[33] Duvaud S., Gabella C., Lisacek F., Stockinger H., Ioannidis V., Durinx C. (2021). Expasy, the Swiss Bioinformatics Resource Portal, as designed by its users. Nucleic Acids Res..

[34] Letunic I., Khedkar S., Bork P. (2021). SMART: Recent updates, new developments and status in 2020. Nucleic Acids Res..

[35] Zheng W., Zhang C., Li Y., Pearce R., Bell E.W., Zhang Y. (2021). Folding non-homologous proteins by coupling deep-learning contact maps with I-TASSER assembly simulations. Cell Rep. Methods.

[36] Burge C., Karlin S. (1997). Prediction of complete gene structures in human genomic DNA. J. Mol. Biol..

[37] Dürig N., Jude R., Holl H., Brooks S.A., Lafayette C., Jagannathan V., Leeb T. (2017). Whole genome sequencing reveals a novel deletion variant in the KIT gene in horses with white spotted coat colour phenotypes. Anim. Genet..

[38] Kent W.J. (2002). BLAT—The BLAST-Like Alignment Tool. Genome Res..

[39] Torella A., Zanobio M., Zeuli R., del Vecchio Blanco F., Savarese M., Giugliano T., Garofalo A., Piluso G., Politano L., Nigro V. (2020). The position of nonsense mutations can predict the phenotype severity: A survey on the DMD gene. PLoS ONE.

[40] Attanasio C., David A., Neerman-Arbez M. (2003). Outcome of donor splice site mutations accounting for congenital afibrinogenemia reflects order of intron removal in the fibrinogen alpha gene (FGA). Blood.

[41] Ma S.L., Vega-Warner V., Gillies C., Sampson M.G., Kher V., Sethi S.K., Otto E.A. (2015). Whole exome sequencing reveals novel PHEX splice site mutations in patients with hypophosphatemic rickets. PLoS ONE.

[42] Hori T., Fukao T., Murase K., Sakaguchi N., Harding C.O., Kondo N. (2013). Molecular basis of two-exon skipping (exons 12 and 13) by c.1248+5g>a in OXCT1 gene: Study on intermediates of OXCT1 transcripts in fibroblasts. Hum. Mutat..

[43] Holl H., Brooks S.A., Bailey E. (2010). De novo mutation of KIT discovered as a result of a non-hereditary white coat colour pattern. Anim. Genet..

[44] Richards S., Aziz N., Bale S., Bick D., Das S., Gastier-Foster J., Grody W.W., Hegde M., Lyon E., Spector E. (2015). Standards and guidelines for the interpretation of sequence variants: A joint consensus recommendation of the American College of Medical Genetics and Genomics and the Association for Molecular Pathology. Genet. Med..

